# Geographical origin orchestrates acteoside accumulation in *Rehmannia glutinosa* via coordinated transcriptomic and metabolomic reprogramming

**DOI:** 10.3389/fpls.2026.1786711

**Published:** 2026-04-02

**Authors:** Tingting Zhang, Jin Wen, Rongjun Zhang, Bao Zhang, Caixia Xie, Zeyu Zheng, Wencai Ma, Mingjie Li, Zhongyi Zhang, Long Lu, Li Gu

**Affiliations:** 1 College of Bee Science and Biomedicine, Fujian Agriculture and Forestry University, Fuzhou, China; 2 State Key Laboratory for Quality Ensurance and Sustainable Use of Dao-di Herbs, Beijing, China; 3 Key Laboratory of Ministry of Education for Genetics, Breeding and Multiple Utilization of Crops, College of Agriculture, Fujian Agriculture and Forestry University, Fuzhou, China; 4 School of Pharmacy, Henan University of Chinese Medicine, Zhengzhou, China

**Keywords:** acteoside, geographical origin, metabolomics, *Rehmannia glutinosa*, transcriptomics

## Abstract

**Introduction:**

The geo-authenticity of medicinal plants, exemplified by *Rehmannia glutinosa*, is largely attributed to environmentally driven variation in bioactive compounds; yet, the underlying systemic molecular mechanisms remain elusive.

**Methods:**

This study employed an integrated approach combining targeted quantitative analysis, transcriptomics, and metabolomics to dissect how geographical origin shapes the medicinal quality of *Rehmannia glutinosa*, using two cultivars ('Wen 85-5' and 'Jin Jiu') sourced from two distinct production regions (Henan and Hebei).

**Results:**

Targeted quantification confirmed origin-specific accumulation patterns of key bioactive compounds, most notably a significantly higher acteoside content in roots from the Henan origin. Multi-omics profiling revealed a conserved core molecular response to geographical origin, involving 894 common differentially expressed genes and 443 common differentially abundant metabolites enriched in hormone signaling, primary metabolism, and specialized biosynthesis pathways. While the response amplitude was genotype-dependent (stronger in 'Wen 85-5'), its fundamental architecture was consistent. Crucially, we identified a coordinated upregulation of the entire acteoside biosynthetic network in Henan-sourced roots. This was evidenced by the concerted induction of key structural genes (*PAL, C4H, 4CL, TyDC, UGT*) across both phenylpropanoid and tyrosine-derived branches, coupled with elevated levels of pathway intermediates.

**Conclusion:**

This study elucidates that the geo-authenticity of *Rehmannia glutinosa* arises from an origin-triggered, systemic reconfiguration of interconnected transcriptional and metabolic networks. The identified core regulatory network provides a mechanistic framework for understanding quality formation and paves the way for molecular-assisted cultivation and breeding strategies.

## 1 Introduction

*Rehmannia glutinosa* (*R. glutinosa*), a medicinally (1) significant species in the Scrophulariaceae family, has been utilized in traditional Chinese medicine (TCM) for thousands of years (Wang et al., 2025). Recorded as a “top-grade” herb in the ancient classic “Shennong’s Herba”, it is highly valued for nourishing Yin and tonifying the kidney according to TCM theory (Zhang et al., 2008). Modern pharmacological studies have revealed a spectrum of bioactive properties for *R. glutinosa*, including hypoglycemic (Qin et al., 2018), antioxidant (Li et al., 2020), anti-inflammatory (Liu et al., 2012), immunoenhancing (Huang et al., 2013), antidepressant (Wang et al., 2018), anti-cancer (Lu et al., 2022), and anti-aging effects (Bai et al., 2018). These activities are attributed to its rich profile of secondary metabolites, particularly iridoid glycosides (e.g., catalpol and harpagide) and phenylethanoid glycosides (e.g., acteoside and isoacteoside), which constitute the primary material basis for its pharmacological efficacy (He et al., 2011; Li et al., 2015; Zhang et al., 2008).

However, the utilization of *R. glutinosa* faces a challenge common to many medicinal plants, namely, substantial variation in the content of these critical bioactive compounds associated with geographical origin (Long et al., 2023; Pacholczyk-Sienicka et al., 2024; Zhang et al., 2024). Extensive phytochemical studies confirm that growth environment profoundly influences the abundance of active constituents (Kadu et al., 2012; Pacholczyk-Sienicka et al., 2024; Zhang et al., 2025). This chemotypic variation is considered a manifestation of plant adaptation to combinatorial environmental stresses, which are driven by factors such as soil composition (Flores et al., 2021), climatic conditions (Li et al., 2024a; Sarker and Oba, 2018), and agricultural practices (Juroszek et al., 2009; Tavarini and Angelini, 2013). These external factors are known to trigger internal transcriptional and metabolic reprogramming (Cao et al., 2025; Xu et al., 2025).

In recent years, transcriptomic approaches have begun to uncover the genetic basis for the biosynthesis of several active compounds in *Rehmannia* (Li et al., 2024b; Wang et al., 2021, Wang et al., 2017). For instance, key enzyme genes involved in the iridoid glycoside pathway (e.g., catalpol biosynthesis) have been identified and linked to terpenoid production (Kang et al., 2022). Similarly, enzymes critical for phenylethanoid glycoside synthesis, such as RgCuAO, RgPAR, and RgUGT in the salidroside pathway, have been characterized and reconstituted in yeast (Yang et al., 2024). However, these studies are largely conducted under controlled conditions. They therefore provide limited insight into how complex, natural environmental signals systemically remodel global regulatory networks to coordinate the accumulation of specific compounds in field-grown plants. The molecular mechanisms by which geo-origin signals integrate and regulate multiple interconnected biosynthetic pathways *in situ* remain unclear.

To address this gap, integrated multi-omics approaches have emerged as a powerful strategy (Wang et al., 2024; Wu et al., 2025). Recent studies have demonstrated their utility in deciphering geo-authenticity: in *Salvia miltiorrhiza*, multi-omics analysis combined with genetic validation revealed how transcription factors (e.g., SmWRKY40) mediate phenolic acid accumulation in roots from a geo-authentic region, establishing a genetic-environmental co-regulatory model (Yu et al., 2025); in *Alpinia oxyphylla*, integrated transcriptomic and metabolomic profiling identified key genes and biomarkers associated with quality differences between authentic and non-authentic producing areas (Pan et al., 2025). These cases underscore that multi-omics integration can effectively decipher systemic metabolic reprogramming in response to diverse environmental signals, providing a systems-level perspective essential for elucidating the molecular basis of geo-authenticity.

Building on this rationale, the present study employed two representative cultivars, ‘Wen 85-5’ and ‘Jin Jiu’, which exhibit measurable genetic and phenotypic differentiation (Li et al., 2018). An integrated transcriptomic and metabolomic analysis of *R. glutinosa* was then conducted to dissect the molecular basis of origin-specific variation. By comparing these two distinct cultivars from two production regions (Henan and Hebei), we aim to: 1) identify the conserved core of origin-responsive genes and metabolites across genetic backgrounds; 2) integrate these molecular layers through correlation and pathway-based association analysis to construct potential regulatory links; and 3) elucidate how geo-origin effects converge on specific metabolic pathways, thereby providing a mechanistic explanation for the differential accumulation of pivotal active compounds. Our findings are expected to provide novel insights into the molecular determinants of medicinal plant quality, offering a robust framework for the sustainable cultivation and quality optimization of *R. glutinosa*.

## 2 Materials and methods

### 2.1 Plant material

Two cultivars of *Rehmannia glutinosa* Libosch., ‘Wen 85-5’ and ‘Jin Jiu’, were used in this study. Both cultivars were bred by the Wen County Institute of Agricultural Sciences (Wen County, Henan, China). The plants were grown in two production regions: Wen County, Henan Province (34.9396°N, 113.0807°E) and Anguo County, Hebei Province (38.41899°N, 115.32709°E). Mature tuberous roots were harvested from one-year-old plants on October 28, between 9:00 AM and 11:00 AM. For each cultivar-origin combination, fresh root samples collected from multiple plants were immediately frozen in liquid nitrogen and stored at -80 °C for subsequent transcriptomic and metabolomic analyses. Three independent replicates were analyzed. Each replicate consisted of a composite sample pooled from several individual plants.

### 2.2 Environmental data

To characterize the environmental differences between the two production regions, the climatic and soil data for the sampling sites in Wen County (Henan) and Anguo County (Hebei) were collected. Nineteen climatic variables were obtained from the WorldClim database (https://www.worldclim.org) at a spatial resolution of 30 seconds (~1 km). Soil factors for the 0–30 cm surface layer were extracted from the World Soil Database (http://www.crensed.ac.cn/). The detailed environmental profiles of the two sites are provided in **Supplementary Table 1**.

### 2.3 Determination of key bioactive ingredients

The contents of 2′−acetylacteoside, acteoside, isoacteoside, salidroside, catalpol, and ajugol in *R. glutinosa* samples were determined with a UPLC-MRM-MS assay using a Waters ACQUITY UPLC coupled with a Xevo TQ-XS MS (Waters Corporation, Milford, MA). Reference standards of the six analytes (Desite Bio−Technology, Chengdu, China) were dissolved in methanol−water (1:1, v/v) to prepare stock and working solutions for calibration. Fresh *R. glutinosa* roots were collected, dried, and pulverized into a fine powder (passed through a 65-mesh sieve). The powdered sample (0.1 g) was extracted twice with 1 mL of methanol−water (1:1, v/v) under ultrasonication (30 min, 40 kHz). After centrifugation and filtration, the extract was analyzed on an Agilent ZORBAX SB−C18 column (100 mm × 2.1 mm, 1.8 μm) with a gradient of 0.1% formic acid in water (A) and acetonitrile (B) at 0.3 mL/min. Detection was performed on a triple−quadrupole mass spectrometer equipped with an electrospray ionization source in negative ion mode, using multiple reaction monitoring (MRM) with the transitions summarized in **Table 1**.

**Table 1:** MRM parameters for the target compounds.

Compound	Retention time (min)	Precursor ion (m/z)	Product ion (m/z)	Cone voltage (V)	Collision energy (eV)
2’-Acetylacteoside	6.10	665.2	160.9	30	20
Acteoside	5.32	623.2	160.9	30	20
Isoacteoside	5.60	623.2	160.9	30	20
Salidroside	3.18	299.1	119.1	30	20
Catalpol	1.04	361.1	169.1	40	20
Ajugol	2.54	347.1	167.1	50	20

### 2.4 Untargeted metabolomic analysis

Metabolite extraction from tissue samples (100 mg) was conducted by grinding under liquid nitrogen, followed by resuspension in prechilled 80% methanol. After vortexing and incubation on ice for 5 min, the homogenate was centrifuged at 15, 000 g and 4 °C for 20 min. The supernatant was diluted with LC-MS grade water to a final concentration of 53% methanol, recentrifuged under identical conditions, and the resulting supernatant was subjected to UHPLC-MS/MS analysis.

Chromatographic separation was performed on a Vanquish UHPLC system coupled to a Q Exactive^TM^ HF or HF-X mass spectrometer (Thermo Fisher, Germany) using a Hypersil Gold column (100 × 2.1 mm, 1.9 μm) with a 12-min gradient of methanol and 0.1% formic acid in water at 0.2 mL/min. Mass spectrometry operated in both polarities with a spray voltage of 3.5 kV and capillary temperature of 320 °C. Raw data were processed via XCMS for peak alignment and quantification. Metabolites were putatively annotated by matching MS1 spectra against spectral databases with a mass deviation of 10 ppm. Annotation confidence was elevated for metabolites with MS/MS spectral support. After blank subtraction, data were normalized, and metabolites with a CV > 30% in QC samples were excluded. Statistical and bioinformatic analyses included principal component analysis (PCA), partial least squares discriminant analysis (PLS-DA), *t*-tests, and Kyoto Encyclopedia of Genes and Genomes (KEGG) pathway enrichment, with visualization performed in R using packages such as ggplot2, pheatmap, and corrplot.

### 2.5 Illumina transcriptomic sequencing

RNA integrity was verified using an Agilent Bioanalyzer 2100 system with the RNA Nano 6000 Assay Kit. Sequencing libraries were constructed from total RNA, in which mRNA was isolated using poly-T oligo-attached magnetic beads and fragmented. First-strand cDNA was synthesized with random hexamers and M-MuLV Reverse Transcriptase, followed by second-strand synthesis with DNA Polymerase I and RNase H. After end repair, adenylation, and adapter ligation, cDNA fragments of 370–420 bp were selected and amplified by PCR. Library quality was confirmed using the Agilent Bioanalyzer. Cluster generation was conducted on a cBot Cluster Generation System with the TruSeq PE Cluster Kit, and paired-end sequencing (150 bp) was performed on an Illumina Novaseq platform.

Following quality control with fastp to remove adapter-containing, poly-N, and low-quality reads, clean data were obtained, and Q20, Q30, and GC content were calculated. Subsequent alignment of clean reads to the reference genome was performed using Hisat2 v2.0.5, which incorporated splice junction information to improve mapping accuracy. Transcript assembly and novel transcript prediction were conducted using StringTie, after which gene expression levels were quantified via featureCounts and reported in FPKM values to account for sequencing depth and gene length.

### 2.6 Reverse transcription-quantitative PCR analysis

To validate the RNA-seq data, eight differentially expressed genes were r selected for SYBR Green-based RT-qPCR analysis. Gene-specific primers were designed using Primer Premier 5 with *RgTIP4* as the internal control. The 20 μL reaction system contained 10 μL of 2× ChamQ Universal SYBR qPCR Master Mix, 0.4 μL of each primer, 1 μL cDNA template, and 8.2 μL RNase-free water. Amplification was performed on a CFX96 Real-Time PCR Detection System under the following conditions: initial denaturation at 95 °C for 10 min, followed by 40 cycles of 95 °C for 10 s, 60 °C for 30 s, and 72 °C for 30 s. All reactions were conducted with three biological and three technical replicates. Primer sequences used for gene expression analysis are listed in **Supplementary Table 2**.

## 3 Results

### 3.1 Differential accumulation of key bioactive compounds in relation to geographical origin

**Figure 1:**
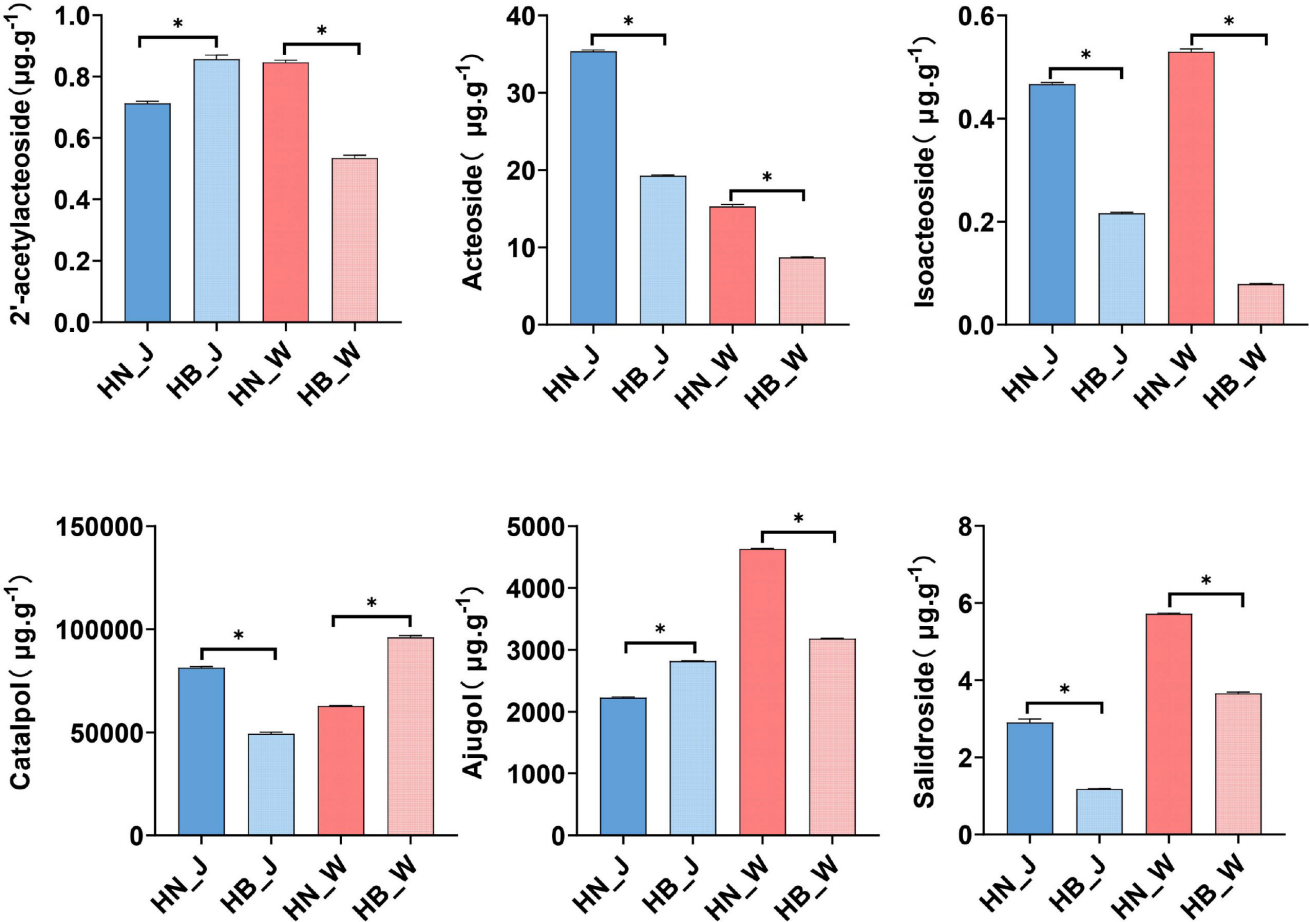
Bioactive constituents of ‘Wen 85-5’ (W) and ‘Jin Jiu’ (J) cultivated in Henan (HN) and Hebei (HB) provinces. Contents of six key bioactive compounds in tuberous roots. Data are mean ± SD (n = 3). For each compound within a cultivar, asterisks indicate significant differences among geographical origins (*p < 0.05, one-way ANOVA).

To assess the direct impact of geographical origin on the medicinal quality of *R. glutinosa*, a targeted analysis was conducted to quantify six key bioactive constituents in the tuberous roots of cultivars ‘Wen 85-5’ and ‘Jin Jiu’ cultivated in Henan and Hebei provinces. The analyzed compounds encompass two major classes: phenylethanoid glycosides (2′-acetylacteoside, acteoside, isoacteoside, and salidroside) and iridoid glycosides (catalpol and ajugol). Analysis revealed distinct and regular patterns in the accumulation of these key components (**Figure 1**). Specifically, the phenylethanoid glycosides acteoside and isoacteoside, along with salidroside, showed a coordinated accumulation pattern, with their contents consistently higher in samples from Henan compared to those from Hebei within the same cultivar (e.g., the content of acteoside in ‘Jin Jiu’ from Henan was approximately 1.8-fold higher than in its Hebei counterpart). In contrast, 2′-acetylacteoside, catalpol, and ajugol displayed divergent accumulation profiles. Notably, the accumulation of the core iridoid glycoside catalpol exhibited a clear cultivar-by-origin interaction, with the highest content observed in ‘Wen 85-5’ from Hebei and in ‘Jin Jiu’ from Henan. These chemical profiling results demonstrate that geographical origin significantly alters the accumulation of key bioactive compounds in *R. glutinosa* roots, and that this influence varies across different chemical classes. To uncover the system-level molecular regulatory network underlying these chemical phenotypic disparities, integrated transcriptomic and metabolomic analyses were subsequently conducted.

### 3.2 Transcriptomic responses to geographical origin

To elucidate the molecular mechanisms underlying the response of *R. glutinosa* to different geographical origins, transcriptome analysis was performed. Following the filtration of low-quality reads, the number of clean reads per library ranged from 39, 783, 098 to 55, 530, 582. Over 90% of the clean reads in each library were successfully mapped to the reference genome of *R. glutinosa*, confirming the suitability of the data for subsequent analysis. The high base call accuracy rate (Q30 > 95.11%–97.91%) and stable GC content (42.95%–44.67%) further verified the reliability and high quality of the sequencing data.

**Figure 2:**
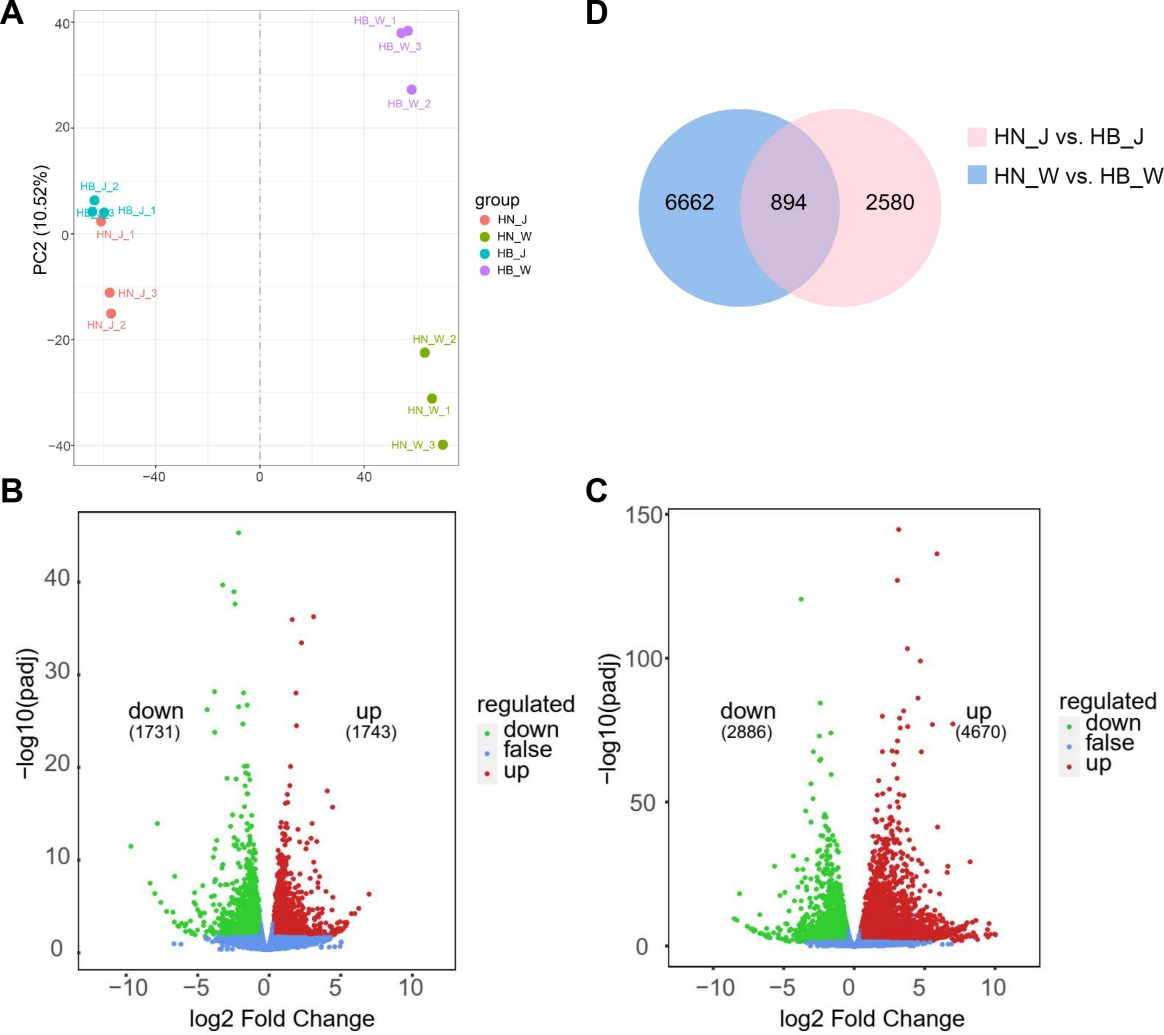
Transcriptomic profiles of *R. glutinosa* cultivars. **(A)** Principal component analysis (PCA) of samples from the four experimental groups. **(B, C)** The volcano plots showing differentially expressed genes (DEGs) identified in the HN_J vs. HB_J **(B)** and HN_W vs. HB_W **(C)**. **(D)** Venn diagram illustrating the overlap of DEGs between the two comparisons (J vs. W).

Principal component analysis (PCA) revealed distinct separation among samples based on both geographical origin and cultivar at the transcriptome level (**Figure 2A**). Notably, a clear spatial divergence was observed between the ‘Wen 85-5’ samples from Henan (HN_W) and Hebei (HB_W). In contrast, the ‘Jin Jiu’ samples from the two origins (HN_J and HB_J) formed a tight cluster with considerable overlap, indicating a more similar transcriptional state between origins for this cultivar.

To identify genes significantly affected by geographical origin, differentially expressed genes (DEGs) were analyzed (**Supplementary Table 3**). For the HN_J vs. HB_J comparison, 3, 474 significant DEGs were screened, comprising 1, 743 upregulated and 1, 731 downregulated genes (**Figure 2B**). In the HN _W vs. HB_W comparison, a total of 7, 556 DEGs were identified, with 4, 670 being upregulated and 2, 886 downregulated (**Figure 2C**). Across these inter-origin comparisons, the number of upregulated DEGs was higher than that of downregulated DEGs. Furthermore, both the upregulated and downregulated DEG counts were higher in ‘Wen 85-5’ than in ‘Jin Jiu’. Subsequent Venn diagram analysis identified 894 common DEGs shared between the two comparison groups (**Figure 2D**), representing a conserved transcriptional core responsive to geographical variation.

**Figure 3:**
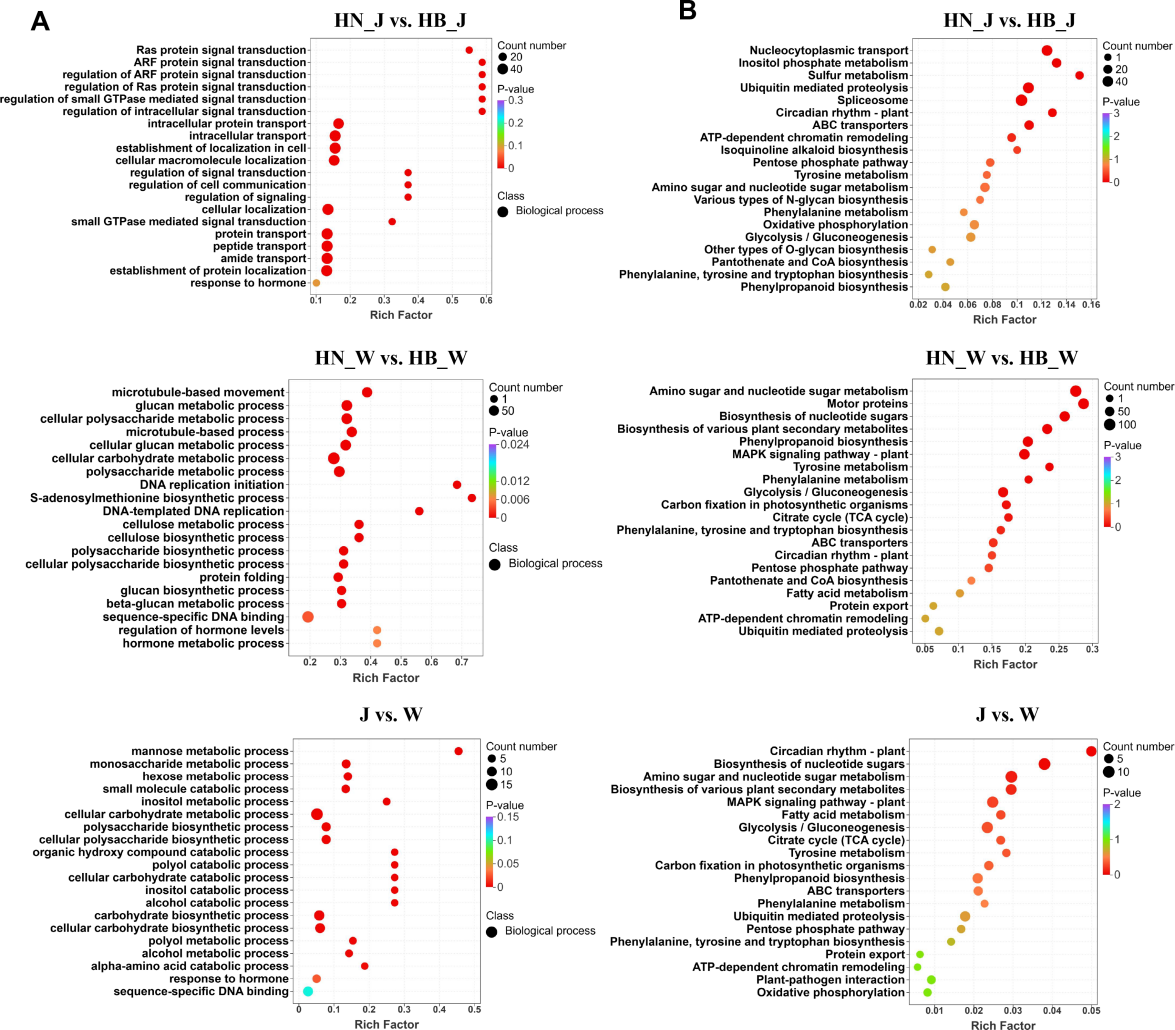
GO and KEGG pathway enrichment analysis of DEGs. The significantly enriched Gene Ontology (GO) terms **(A)** and Kyoto Encyclopedia of Genes and Genomes (KEGG) pathways **(B)** for differentially expressed genes (DEGs) identified in the three comparison groups (HN_J vs. HB_J, HN_W vs. HB_W, and J vs. W).

To elucidate the biological functions of DEGs in response to geographical origins, Gene Ontology (GO) and KEGG pathway enrichment analyses were performed for the inter-origin comparison groups (**Figures 3A, B**). In the HN_J vs. HB_J comparison, GO analysis revealed significant enrichment in terms including Ras proten signal transduction, ARF protein signal transduction, small GTPase mediated signal transduction, regulation of signal transduction, and response to hormone. This indicates that the primary molecular response to geo-environmental differences involves the perception and relay of external signals. The corresponding KEGG analysis showed enrichment in pathways such as Ubiquitin mediated proteolysi, Circadian rhythm-plant, Tyrosine metabolism, Phenylalanine, tyrosine and tryptophan biosynthesis, and Phenylpropanoid biosynthesis. For the HN_W vs. HB_W comparison, GO analysis displayed prominent enrichment for terms related to microtubule-based movement, microtubule-based process, cellulose biosynthetic process, sequence-specific DNA binding, regulation of hormone levels, and hormone metabolic process. KEGG analysis for this group exhibited enrichment in pathways including Biosynthesis of various plant secondary metabolites, Phenylpropanoid biosynthesis, MAPK signaling pathway- plant, Tyrosine metabolism, Phenylalanine, tyrosine and tryptophan biosynthesis, and Circadian rhythm-plant.

Analysis of the common DEGs shared between the two cultivars (J vs. W) showed that the GO terms were prominently enriched for monosaccharide/hexose metabolic process, cellular carbohydrate metabolic process, carbohydrate biosynthetic process, response to hormone, and sequence-specific DNA binding (**Figure 3A**). The KEGG analysis for these common DEGs exhibited enrichment in pathways including Circadian rhythm-plant, MAPK signaling pathway-plant, Tyrosine metabolism, Phenylpropanoid biosynthesis, and Phenylalanine, tyrosine and tryptophan biosynthesis (**Figure 3B**). Collectively, the enrichment analyses indicate that geographical origin induces a conserved transcriptional reprogramming in *R. glutinosa* roots. This reprogramming is characterized by the co-enrichment of environmental signal perception pathways (e.g., circadian rhythm), signal transduction and hormonal response pathways, and culminates in the systemic up-regulation of the core biosynthetic pathways for specialized metabolites, particularly those related to phenylpropanoid and tyrosine metabolism.

### 3.3 Metabolic characterization in response to geographical origin

**Figure 4:**
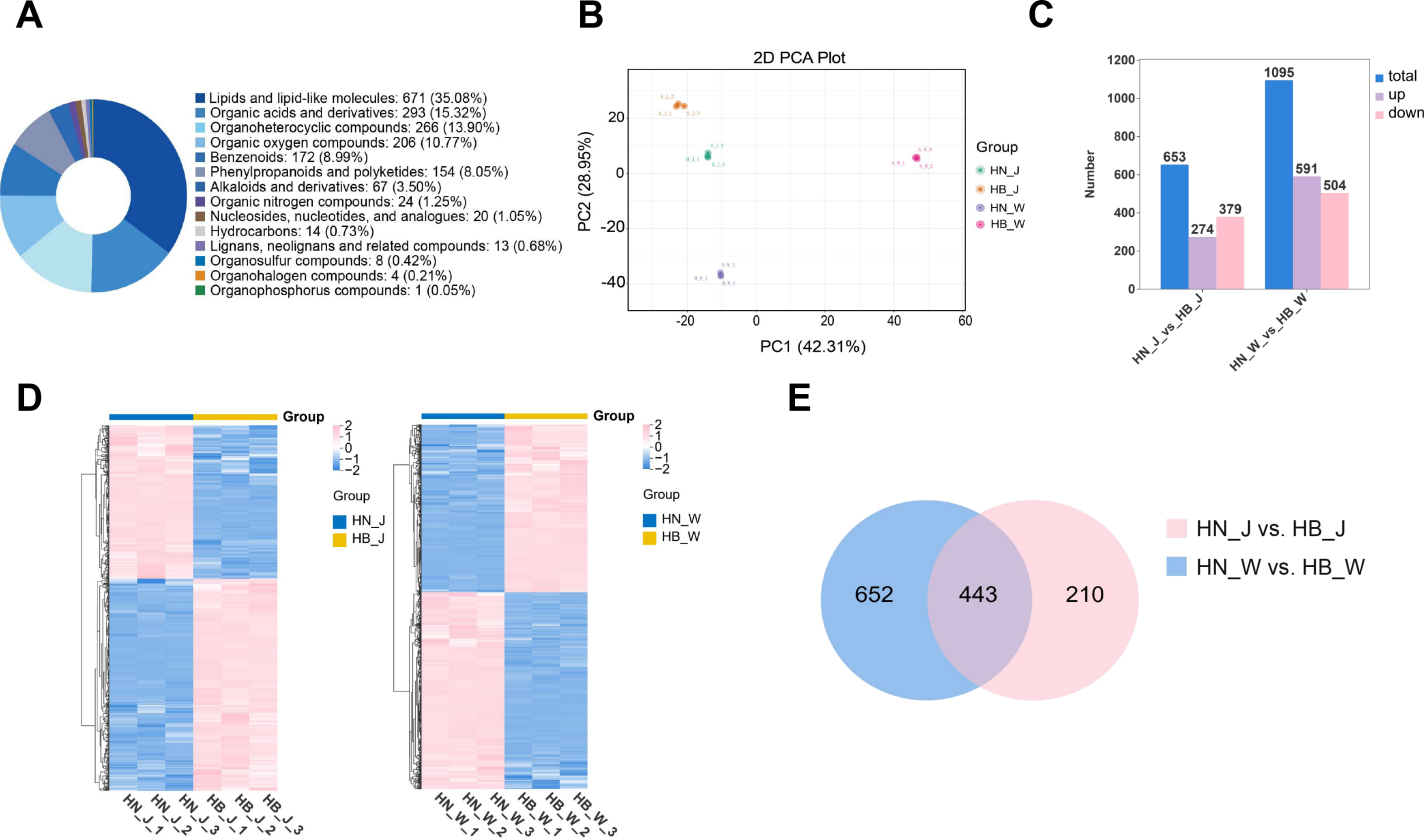
Metabolomic profiles of R. glutinosa cultivars. **(A)** Composition of metabolite classes in tuberous roots. **(B)** PCA analysis of metabolic profiles. **(C)** Numbers of differentially abundant metabolites (DAMs) in HN_J vs. HB_J and HN_W vs. HB_W comparisons. **(D)** Hierarchical clustering heatmap of metabolite variations in HN_J vs. HB_J and HN_W vs. HB_W. **(E)** Venn diagram showing common DAMs between HN_J vs. HB_J and HN_W vs. HB_W.

A total of 1, 913 metabolites were identified across all *R. glutinosa* samples and classified into 14 major categories (**Figure 4A**, **Supplementary Table 4**). These included lipids and lipid-like molecules (671), organic acids and derivatives (292), organoheterocyclic compounds (266), organic oxygen compounds (206), benzenoids (172), phenylpropanoids and polyketides (154), alkaloids and derivatives (67), organic nitrogen compounds (24), nucleosides, nucleotides, and analogues (20), hydrocarbons (14), lignans, neolignans, and related compounds (13), organic sulfur compounds (8), organohalogen compounds (4), and Organophosphorus compounds (1). To globally assess the variation in metabolite profiles, unsupervised principal component analysis (PCA) was performed. The PCA score plot (**Figure 4B**) revealed distinct clustering patterns. Samples from the two cultivars were clearly separated along the principal components, indicating that the genotype (cultivar) substantially shapes the metabolic signature of *R. glutinosa.* Within the ‘Wen 85-5’ cultivar, samples from Henan (HN_W) and Hebei (HB_W) were distributed in the first and third quadrants, respectively, with a considerable distance between them, reflecting a high sensitivity of this cultivar’s metabolome to the geographical origin. In contrast, samples of the ‘Jin Jiu’ cultivar from both origins clustered tightly in the second quadrant, suggesting its metabolic profile is less influenced by origin and exhibits stronger environmental stability. This observation aligns closely with the trend identified at the transcriptome level, collectively revealing the divergent biological response strategies of the two cultivars to environmental variation.

To identify differentially abundant metabolites (DAMs), an OPLS-DA model combined with Variable Importance in Projection (VIP) was employed. The preliminary screening criteria were set as VIP ≥ 1. Significant DAMs between comparison groups were then identified by combining this with univariate analysis (ANOVA, *P* < 0.05) and a fold-change threshold (FC ≥ 1.5 or FC ≤ 0.67). The results showed that 1, 095 significant DAMs (591 upregulated, 504 downregulated) were identified in the HN_W vs. HB_W comparison, while 653 significant DAMs (379 upregulated, 274 downregulated) were found in the HN_J vs. HB_J comparison (**Figure 4C**). Cluster analysis visualized by a z-score normalized heatmap demonstrated clear hierarchical clustering of these DAMs (**Figure 4D**). A Venn diagram indicated that 443 DAMs were shared between the comparisons (**Figure 4E**).

**Figure 5:**
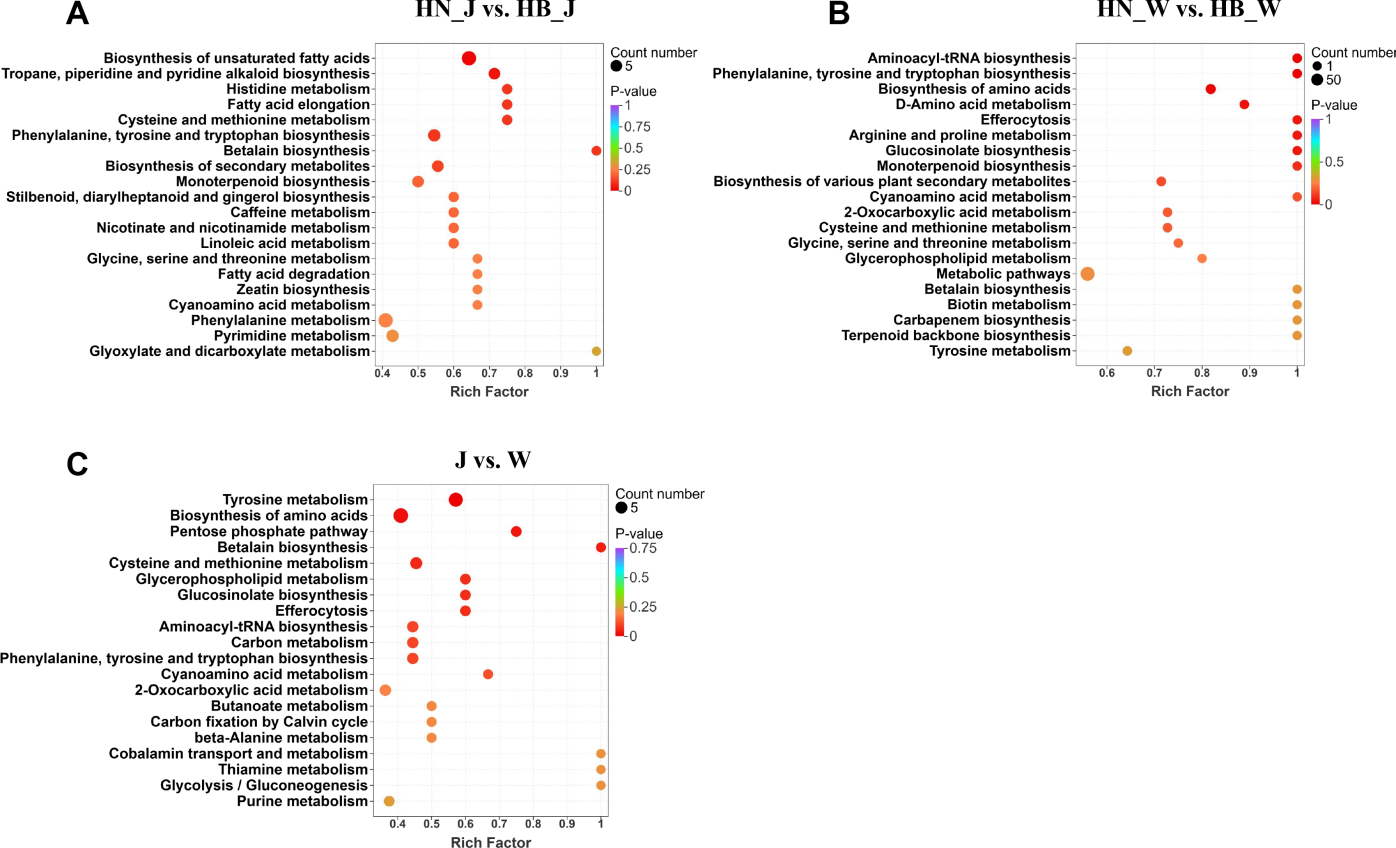
KEGG pathway enrichment analysis of DAMs. KEGG enrichment results for DAMs identified in the comparison groups: **(A)**, HN_J vs. HB_J, **(B)**, HN_W vs. HB_W, and **(C)** J vs. W.

KEGG pathway enrichment analysis was subsequently performed to identify the key metabolic pathways affected by geographical origin. To directly analyze pathway differences between comparison groups, the top 20 most enriched pathways for each group were selected based on enrichment and topological analysis (**Figure 5**). In the HN_J vs. HB_J comparison, the enriched pathways included Phenylalanine, tyrosine and tryptophan biosynthesis, Phenylalanine metabolism, and broader pathways such as Biosynthesis of secondary metabolites. Specific secondary metabolic branches like Stilbenoid, diarylheptanoid and gingerol biosynthesis, Monoterpenoid biosynthesis, and Betalain biosynthesis were also prominent. For the HN_W vs. HB_W comparison, enrichment was similarly observed in the core aromatic amino acid pathway (Phenylalanine, tyrosine and tryptophan biosynthesis) and Tyrosine metabolism. Additionally, Biosynthesis of various plant secondary metabolites was highlighted, along with specific pathways including Monoterpenoid biosynthesis, Betalain biosynthesis, and Terpenoid backbone biosynthesis.

The commonly identified DAMs shared between the two cultivars were enriched in a conserved set of pathways. These primarily included the key precursor-supplying pathways Tyrosine metabolism and Phenylalanine, tyrosine and tryptophan biosynthesis, as well as Biosynthesis of amino acids. Pathways related to specialized metabolism and stress adaptation, such as Cysteine and methionine metabolism, Betalain biosynthesis, and Glucosinolate biosynthesis, were also enriched. Collectively, the KEGG enrichment analysis of differentially abundant metabolites revealed distinct yet overlapping metabolic reprogramming in roots from different geographical origins, prominently involving pathways related to amino acid metabolism and the biosynthesis of diverse secondary metabolites.

### 3.4 Integrated transcriptomic and metabolomic analysis reveals a core regulatory network

**Figure 6:**
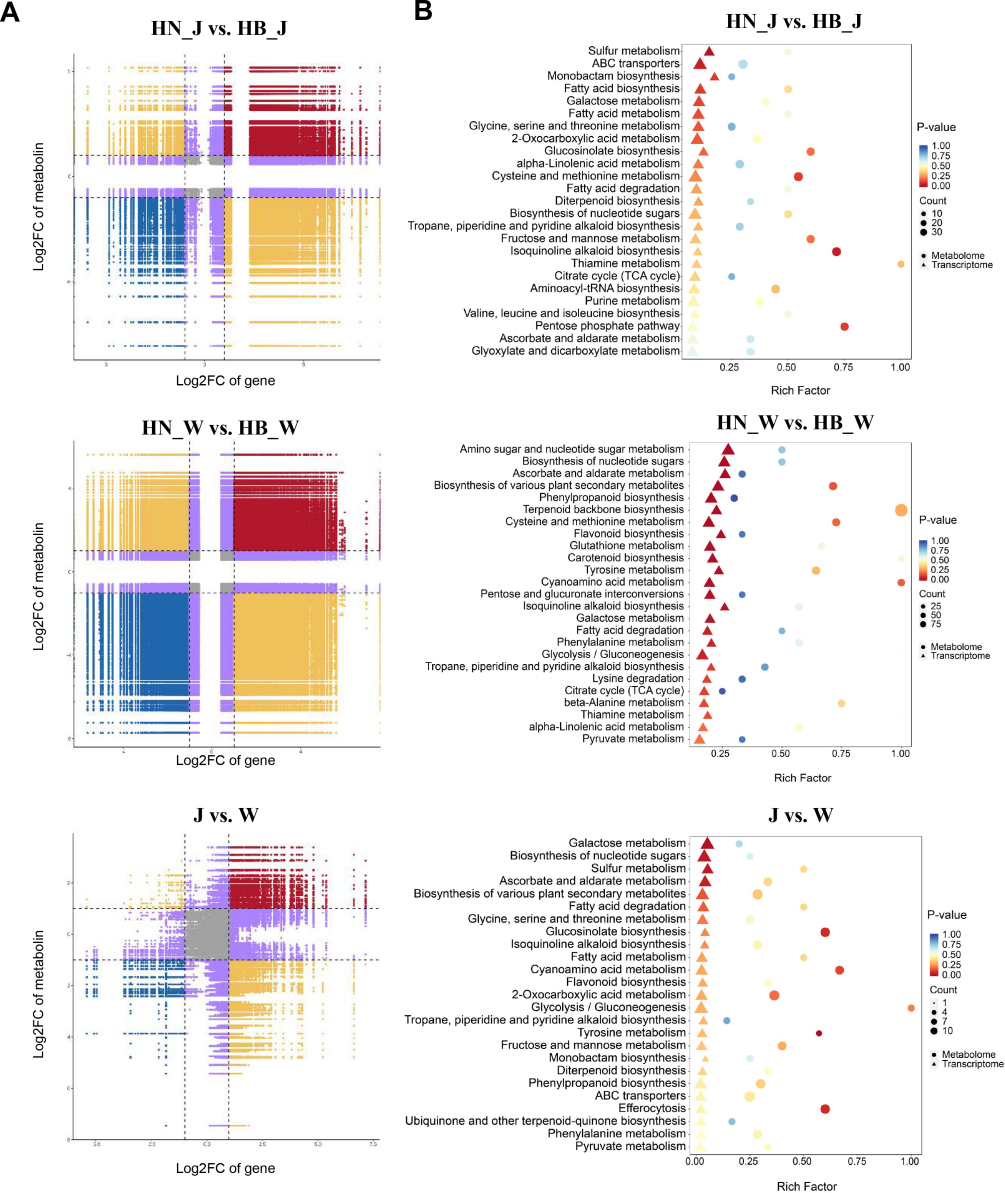
Integrated analysis of transcriptome and metabolome data across production origins. **(A)** Nine-quadrant plot showing correlations between gene expression (log_2_ fold change, X-axis) and metabolite abundance (log_2_ fold change, Y-axis) for three comparison groups. Each point represents a gene-metabolite pair. **(B)** KEGG pathway enrichment analysis integrating transcriptomic and metabolomic data. Enrichment ratio (X-axis) indicates the proportion of genes/metabolites mapped to a pathway, and the Y-axis lists the KEGG pathways. Bubble size reflects the number of mapped molecules, and color represents enrichment significance.

To elucidate the molecular mechanisms linking geographical origin to bioactive compound variation in *R. glutinosa*, an integrated analysis of transcriptomic and metabolomic data was performed. A strategy of cultivar-specific correlation analysis followed by integration was adopted to identify core gene-metabolite regulatory relationships conserved across both ‘Jin Jiu’ and ‘Wen 85-5’. First, Pearson correlation coefficients were calculated between the common DEGs and DAMs within each cultivar’s sample set. These transcriptome-metabolome correlations were visualized using nine-quadrant plots (**Figure 6A**), which categorize gene-metabolite pairs based on their differential expression patterns. The plots revealed several distinct regulatory scenarios: 1) Pairs in quadrants I, II, and IV, where metabolite abundance increased without a corresponding rise in gene expression; 2) Pairs in quadrants VI, VIII, and IX, featuring upregulated genes but non-increasing metabolite levels; and 3) Critically, pairs in quadrants III and VII, where genes and metabolites exhibited concordant changes (both upregulated or both downregulated). These strongly correlated pairs likely represent direct or indirect transcriptional regulation of metabolite accumulation and formed the core set for constructing a putative regulatory network. To ensure robustness, subsequent analysis focused on gene-metabolite pairs that demonstrated highly consistent and significant correlations (|r| > 0.8, *p* < 0.05, with the same correlation direction) in both cultivars.

This refined set of conserved and cultivar-specific DEGs and DAMs was then mapped to the KEGG pathway database for integrated pathway enrichment analysis (**Figure 6B**). For the ‘Jin Jiu’ cultivar (HN_J vs. HB_J), the integrated pathways were prominently enriched in fundamental stress adaptation and energy metabolism processes, including Sulfur metabolism, ABC transporters, Cysteine and methionine metabolism, and central carbon pathways such as Citrate cycle (TCA cycle) and Pentose phosphate pathway. In contrast, the integrated analysis for the ‘Wen 85-5’ cultivar (HN_W vs. HB_W) revealed an enrichment pattern strongly oriented toward the biosynthesis of specialized metabolites. Key enriched pathways included Phenylpropanoid biosynthesis, Tyrosine metabolism (directly supplying the backbone for acteoside), as well as Flavonoid biosynthesis and Terpenoid backbone biosynthesis. These were coupled with upregulated energy-providing pathways like Glycolysis/Gluconeogenesis. Notably, the integrated pathways derived from the common molecular shared by both cultivars converged on a conserved core network. This core consisted of the essential acteoside-biosynthetic pathways (Phenylpropanoid biosynthesis and Tyrosine metabolism), fundamental stress-response components (Sulfur metabolism and ABC transporters), and the energy-generating Glycolysis/Gluconeogenesis pathway. Collectively, the integrated transcriptome-metabolome KEGG analysis delineates a hierarchical response to geographical origin: a conserved core adaptation network is activated across genotypes, upon which cultivar-specific metabolic reprogramming, particularly pronounced in ‘Wen 85-5’, elaborates to differentially modulate the biosynthesis of a broad spectrum of specialized metabolites.

### 3.5 Coordinated activation of the acteoside biosynthetic network

The biosynthesis of acteoside, a pivotal phenylethanoid glycoside in *R. glutinosa*, originates from two parallel aromatic amino acid precursors: phenylalanine and tyrosine, via the phenylpropanoid and tyrosine-derived pathways, respectively. Integrated transcriptomic and metabolomic analyses provided coherent evidence for a concerted upregulation of this entire biosynthetic network in roots sourced from Henan, which correlated with their higher acteoside content.

**Figure 7:**
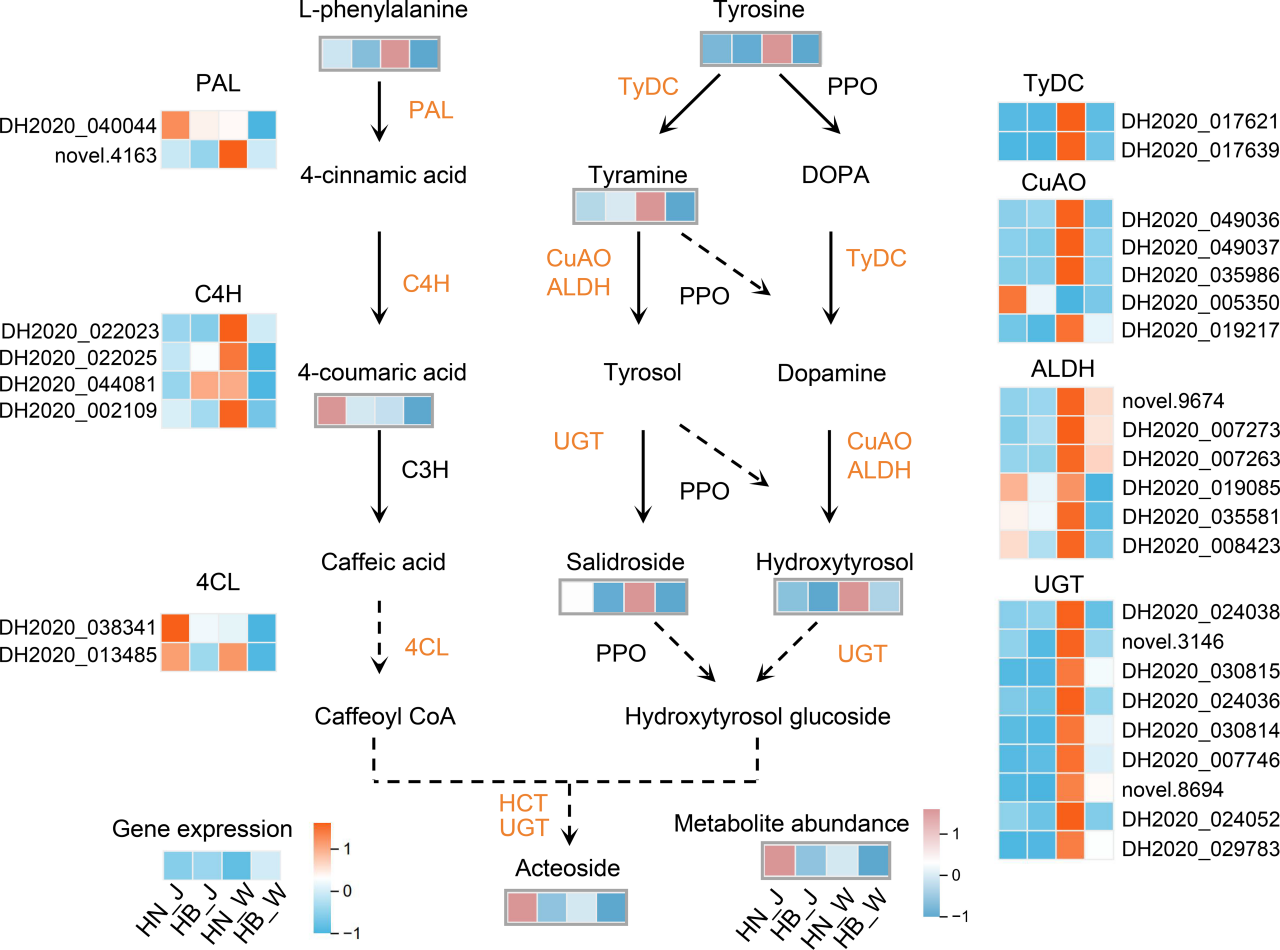
Acteoside biosynthetic pathway with associated gene expression and metabolite profiles. The schematic shows the metabolic route to acteoside, accompanied by a heatmap reflecting the expression of pathway genes and changes in metabolite levels. Enzymes are annotated with their abbreviations: PAL (phenylalanine ammonia-lyase), C4H (cinnamate 4-hydroxylase), C3H (coumarate 3-hydroxylase), 4CL (4-coumarate-CoA ligase), TyDC (tyrosine decarboxylase), CuAO (copper-containing amine oxidase), ALDH (aldehyde dehydrogenase), UGT (UDP-glucosyltransferase), HCT (shikimate O-hydroxycinnamoyltransferase). Relative changes are shown using the color scale.

At the transcriptional level, key structural genes across both precursor pathways were significantly induced in Henan samples (**Figure 7**). In the phenylpropanoid branch responsible for the caffeoyl moiety, a coordinated upregulation was observed for genes encoding phenylalanine ammonia-lyase (PAL), cinnamate 4-hydroxylase (C4H), and 4-coumarate-CoA ligase (4CL). Concurrently, the tyrosine-derived branch for the hydroxytyrosol moiety showed elevated expression of genes involved in the sequential conversion, including tyrosine decarboxylase (TyDC), copper-containing amine oxidase (CuAO), and UDP-glucosyltransferase (UGT). This coordinated transcriptional activation was directly substantiated by corresponding metabolomic profiles. The levels of key pathway intermediates, phenylpropanoid compounds (L-phenylalanine and 4-coumaric acid) and tyrosine-derived metabolites (tyrosine, tyramine, salidroside, and hydroxytyrosol), were consistently and significantly higher in the Henan samples. The congruent enhancement of both gene expression and metabolite abundance across these two parallel biosynthetic streams establishes a systemic molecular mechanism. This mechanism effectively increases metabolic flux toward acteoside, providing a definitive explanation for its elevated accumulation in *R. glutinosa* roots from the Henan origin.

### 3.6 Validation of DEGs in the acteoside biosynthetic pathway by RT-qPCR

**Figure 8:**
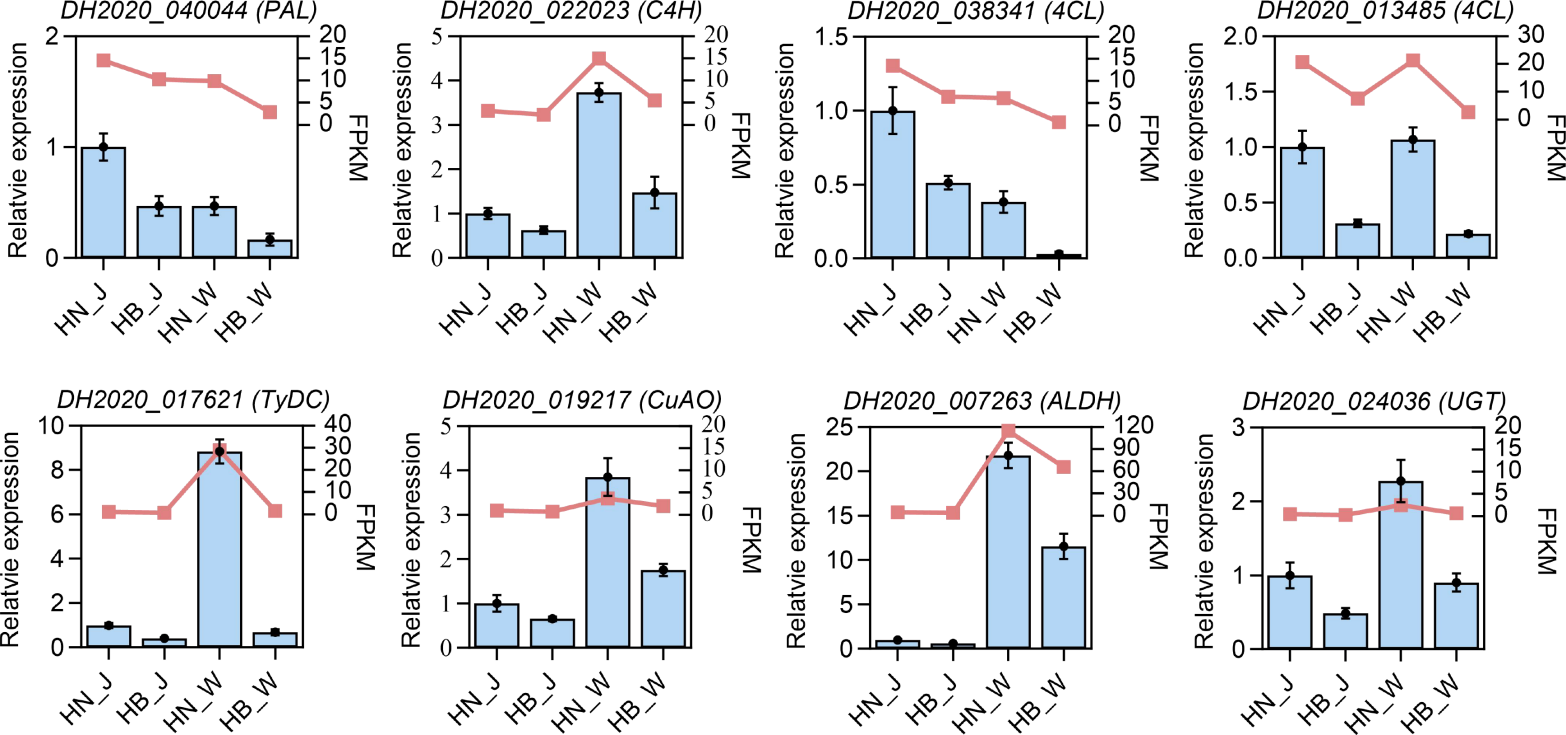
The verification of eight DEGs by RT-qPCR analysis across four groups. Red lines, transcriptome data; bars, corresponding RT-qPCR results.

To verify the accuracy and reproducibility of the RNA sequencing results, the expression levels of seven DEGs implicated in the acteoside biosynthetic pathway were examined using quantitative real-time PCR (RT-qPCR). The selected genes included: *PAL* (DH2020_040044), *C4H* (DH2020_022023), *4CL* (DH2020_038341 and DH2020_013485), *TyDC* (DH2020_017621), *CuAO* (DH2020_019217), *ALDH* (DH2020_007263), and *UGT* (DH2020_024036). The relative expression trends of these seven key genes across different geographical origins (**Figure 8**), as determined by RT-qPCR, were consistent with the patterns observed in the transcriptome sequencing data.

## 4 Discussion

The substantial influence of geographical origin on the bioactive constituents of medicinal plants represents a central scientific question underlying the traditional concept of “Daodi” (geo-authenticity) in Chinese herbal medicine. Substantial evidence indicates that this influence is widespread across diverse medicinal species and plays a decisive role in determining their pharmacological potency (Long et al., 2023; Shang et al., 2023; Zou et al., 2024). For instance, *Atractylodes lancea* exhibits distinct chemotypes, characterized by differential accumulation of markers such as β-eudesmol and atractylodin, across its northern and southern production regions (Zhang et al., 2023). For *R. glutinosa*, regional disparity is similarly a critical determinant of quality. Our targeted quantitative analysis (**Figure 1**) clearly demonstrates systematic differences in the accumulation of core bioactive compounds, including acteoside and catalpol, between roots sourced from Henan and Hebei provinces, with acteoside derivatives consistently showing higher abundance in Henan samples. Therefore, elucidating how the composite environmental signals of a specific origin mechanistically regulate the metabolic phenotype of *R. glutinosa* at the molecular level is essential for understanding the foundation of its quality formation.

Environmental heterogeneity, encompassing variations in light, temperature, water, and soil conditions, is increasingly recognized as a key driver of secondary metabolite accumulation in medicinal plants. These abiotic factors activate plant stress-responsive mechanisms, thereby reshaping secondary metabolic networks. This phenomenon is well-documented across diverse medicinal species. Integrated multi-omics analyses have revealed how specific environmental factors drive metabolic reprogramming: drought stress in *Dendrobium nobile* (Lv et al., 2025), altitudinal adaptation in *Phlomoides rotata* (Wu et al., 2025), and climatic factors such as precipitation and temperature in *Cistanche deserticol*a (Li et al., 2019) all significantly influence the accumulation of key secondary metabolites. Focusing on *Rehmannia glutinosa*, He et al. (2024) systematically elucidated the impact of soil bulk density on tuberous root respiratory metabolism and the accumulation of catalpol and acteoside, identifying the optimal soil bulk density range. Jang et al. (2024) employed metabolomic analysis to delineate metabolic differences between *R. glutinosa* from Korean and Chinese origins, identifying organic acids such as malic acid as origin-specific markers whose abundance varies with environmental conditions. Furthermore, Zhou et al. (2021) demonstrated through metabolomic approaches that wild habitats are more conducive to the accumulation of flavonoids and phenolic acids in *R. glutinosa*. Collectively, these studies demonstrate that origin-associated environmental factors shape the quality of medicinal materials by modulating respiratory metabolism, gene expression, and secondary metabolic networks.

Building on this, our integrated multi-omics analysis shows that the molecular response of *R. glutinosa* to geographical origin may exhibit a degree of genotype dependency. Compared to the ‘Jin Jiu’ cultivar, the ‘Wen 85-5’ cultivar displayed more pronounced transcriptomic and metabolomic reprogramming between different origins, as reflected by its greater numbers of DEGs and DAMs (**Figures 2**, **4**). This suggests the potential existence of differing environmental sensitivities or adaptive strategies among varieties. Despite these quantitative differences between cultivars, our analysis indicates that distinct geographical origins trigger a systematic and shared reprogramming at both the gene expression and metabolite accumulation levels in the tuberous roots. The enrichment of GO terms such as response to hormone, hormone metabolic process and regulation of hormone levels in **Figure 3** suggests that hormones act as pivotal messengers in the plant’s adaptation to origin-specific conditions. This observation aligns with foundational research in other root crop systems. For example, in sweet potato, a well−studied model for tuberous root development, hormones including abscisic acid (ABA), cytokinins, and auxin have been demonstrated to be crucial regulators of root thickening, sink−strength establishment, and carbohydrate accumulation (Nakatani and Komeichi, 1991; Nakatani et al., 1988; Sun et al., 2015). In *R. glutinosa*, multiple hormones, such as indole-3-acetic acid (IAA), ABA, and jasmonic acid (JA), likely serve not only as key modulators of tuber development but also as central hubs that integrate diverse stress signals (e.g., from soil and climate) and subsequently remodel secondary metabolic networks (Zhou et al., 2015).

The distinct climatic and soil conditions between the two origins (**Supplementary Table 1**) provide a plausible environmental basis for the observed molecular reprogramming. Recent studies have demonstrated that climatic factors, particularly temperature and precipitation, are important drivers of secondary metabolite variation in medicinal plants (Bi et al., 2024; Wu et al., 2024). For instance, temperature seasonality and soil available phosphorus have been shown to significantly influence ginsenoside accumulation in *Panax ginseng* (Wang et al., 2024). In our study, the coordinated upregulation of the acteoside biosynthetic pathway in Henan-sourced roots (**Figure 7**) likely represents an adaptive response to the specific environmental conditions of this geo-authentic region.

The specific transcriptional reprogramming is likely also mediated by the action of transcription factors (TFs). Enrichment of TF-related functions such as “sequence-specific DNA binding” within the core response network points to precise transcriptional control (**Figure 3**). In *R. glutinosa*, WRKY family TFs (e.g., RgWRKY37) have been shown to directly bind and activate promoters of key enzyme genes (e.g., *PAL*, *C4H*, *4CL*, *TyDC*) in the acteoside biosynthetic pathway, thereby upregulating its synthesis (Wang et al., 2021). In our study, the coordinated activation of this pathway in Henan samples is consistent with a model in which origin-related signals could influence specific TFs, which in turn may regulate the expression of biosynthetic genes. Together, these findings suggest a potential mechanism through which metabolic flux towards specific metabolites, such as acteoside, might be fine-tuned in response to geographical origin.

The most significant outcome of this origin-driven reprogramming is the systemic and coordinated modulation of the biosynthetic pathways for key bioactive compounds, such as acteoside. The consistently higher accumulation of acteoside in roots from the Henan origin serves as a prime illustration. The biosynthesis of acteoside begins with the shikimate pathway-derived precursors, phenylalanine and tyrosine (Alipieva et al., 2014). Its assembly relies on two parallel and interconnected metabolic streams: the hydroxytyrosol moiety is derived from tyrosine (via tyramine/dopamine), while the caffeoyl moiety is synthesized from phenylalanine through the core phenylpropanoid pathway (Saimaru and Orihara, 2010). Our multi-omics data clearly demonstrate a coordinated upregulation of this entire network in Henan-sourced roots (**Figure 7**). At the transcriptional level, key structural genes from upstream (e.g., *PAL, TyDC*) to downstream (e.g., *4CL, UGT*) were generally upregulated. Concomitantly, at the metabolic level, the abundances of pathway intermediates (e.g., phenylalanine, tyrosine, tyramine, coniferin) were synchronously increased. This “full-chain” activation, spanning from genes to intermediate metabolites, constitutes the direct molecular basis for the significantly elevated acteoside content in *R. glutinosa* from the Henan origin. Notably, the differential expression of distinct 4CL isozymes coincided with elevated acteoside accumulation (Zha et al., 2019), suggesting their involvement in regulating metabolic commitment to acteoside versus other phenolic compounds like lignin or flavonoids.

## 5 Conclusion

This integrated multi-omics study demonstrates that the medicinal quality of *Rehmannia glutinosa* is influenced by geographical origin through coordinated changes at the molecular level. Chemical analysis confirmed distinct accumulation patterns of key bioactive compounds between origins, particularly the higher acteoside content in roots from Henan. Transcriptomic and metabolomic analyses showed that these chemical differences correspond to systematic changes in gene expression and metabolic pathways. A conserved set of 894 differentially expressed genes and 443 differentially abundant metabolites common to both cultivars indicates a shared adaptive response, involving hormone signaling, adjustments in primary metabolism, and the biosynthesis of specialized compounds.

A key mechanistic finding is the coordinated upregulation of the acteoside biosynthetic pathway in roots from Henan. This is supported by the concurrent increased expression of key genes (*PAL, C4H, 4CL, TyDC, UGT*) in both the phenylpropanoid and tyrosine-derived branches, along with raised levels of precursors such as phenylalanine and tyrosine. This multi-level regulation directs metabolic flux toward acteoside synthesis. Although the molecular response was more pronounced in the ‘Wen 85-5’ cultivar than in ‘Jin Jiu’, the activation of this core network was consistently associated with the geographical origin.

In summary, this work shows that the geo-authenticity of *R. glutinosa* results from an origin-dependent, systemic reorganization of its gene expression and metabolic networks. The core molecular components identified here establish a foundation for future strategies in quality evaluation, targeted cultivation, and breeding of this medicinal plant.

## Data Availability

The original contributions presented in the study are publicly available. This data can be found here:Repository NGDC https://ngdc.cncb.ac.cn/gsa, Accession number: CRA040527.
